# Structure and Properties of TiO_2_/nanoTiO_2_ Bimodal Coatings Obtained by a Hybrid PVD/ALD Method on 316L Steel Substrate

**DOI:** 10.3390/ma14164369

**Published:** 2021-08-04

**Authors:** Marcin Staszuk, Daniel Pakuła, Łukasz Reimann, Anna Kloc-Ptaszna, Mirosława Pawlyta, Antonín Kříž

**Affiliations:** 1 Department of Engineering Materials and Biomaterials, Faculty of Mechanical Engineering, Silesian University of Technology, Konarskiego 18A, 44-100 Gliwice, Poland; daniel.pakula@polsl.pl (D.P.); anna.kloc@polsl.pl (A.K.-P.); 2 Materials Research Laboratory, Faculty of Mechanical Engineering, Silesian University of Technology, Konarskiego 18A, 44-100 Gliwice, Poland; lukasz.reimann@polsl.pl (Ł.R.); miroslawa.pawlyta@polsl.pl (M.P.); 3 Department of Materials and Metallurgy Engineering, Faculty of Mechanical Engineering, University of West Bohemia, Univerzitni 22, 30614 Plzen, Czech Republic; kriz@kmm.zcu.cz

**Keywords:** MS-PVD, ALD, hybrid coatings, TiO_2_/nanoTiO_2_ bimodal coatings, S/TEM

## Abstract

This paper presents the synergy of the effect of two surface engineering technologies—magnetron sputtering (MS-PVD) and atomic layer deposition (ALD) on the structure and properties of 316L steel. Recent studies indicate that PVD coatings, despite their thickness of a few micrometers, have many discontinuities and structural defects, which may lead to pitting corrosion after time. Applying an ALD layer to a PVD coating seals its structure and contributes to extending the service life of the coating. Investigations of the structure and morphology of the produced layers were carried out using a scanning electron microscope (SEM) and atomic force microscope (AFM). In addition, the structure of the coatings was investigated on the cross-section using a scanning-transmission electron microscope S/TEM. The tribological properties of the materials studied were determined by the ball-on-disc method. The corrosion resistance of the tested materials was determined by the electrochemical potentiodynamic method by recording the polarization curves of the anodes. Additional information about the electrochemical properties of the tested samples, including the quality, their tightness, and their resistivity, was obtained by electrochemical impedance spectroscopy (EIS). In addition, the main mechanisms of corrosion and tribological wear were determined by SEM observations after corrosion tests and after tribological tests. The study showed that the fabrication of hybrid layers by MS-PVD and ALD techniques allows obtaining coatings with electrochemical properties superior to those of layers fabricated by only one method.

## 1. Introduction

316L austenitic steel has excellent corrosion and mechanical properties, thanks to its chromium and nickel content, which makes it suitable for a variety of industrial and biomedical applications. When considering biomedical applications, many materials exhibit better properties than this steel, e.g., antibacterial properties, and yet this steel is used much more frequently. The main factor that determines the use of 316L steel as a biomedical material is its price and availability. Unfortunately, when used for long periods in conditions that do not allow for regular maintenance (e.g., as an implant in the human body), the passive layer, which determines corrosion resistance, becomes brittle and vulnerable to the external environment. Chloride anions causing pitting corrosion have a particular effect [1]. One of the ways to improve its corrosion resistance and also to increase its biocompatibility is to apply thin films showing the desired properties.

Although coatings on biomedical materials and especially on corrosion-resistant steels have been used for years, their dynamic development has taken place during the last decade. Admittedly, modified PVD methods make it possible to produce coatings that exhibit high abrasion resistance and hardness and good corrosion resistance of the coated parts [2,3]. However, a “perfect” PVD coating that is suitable for a variety of applications and free of structural defects has not yet been produced. On the other hand, although the coatings deposited by the ALD technique on these materials are very thin and do not show very good mechanical properties [4,5], they are characterized by very good anti-corrosion properties [6,7,8,9,10,11,12,13,14].

To obtain a combination of the discussed properties (high corrosion protection and good tribological properties) hybrid coatings are used, which are made by both methods simultaneously [15,16,17]. An example of hybrid PVD + ALD coating, which has been described in [18,19,20], is the CrN-Al_2_O_3_/TiO_2_ coating deposited on steels in various layer arrangement combinations providing the best anti-corrosion properties. In this case, Al_2_O_3_/TiO_2_ layers obtained by the ALD technique tightly cover all imperfections of PVD–CrN coating, providing a barrier for the development of corrosion. In another work, PVD + ALD hybrid coatings consisting of TiAlN/TiN/Al_2_O_3_ and TiCN/Al_2_O_3_ layers were studied [15]. The authors in this work [15] also demonstrated that the ALD layer, or in this case the Al_2_O_3_ phase, seals the PVD layer and thus significantly improves the corrosion resistance of the test substrate.

In the reported studies, this sequence of PVD + ALD layers was usually used [15,21]; this fact is explained by the desire to fill the cavities in the PVD layer. As can be seen from the literature review, the authors have only studied hybrid coatings consisting of different phases. Thus far, there are few studies on coatings consisting of layers of the same phase and obtained by different surface treatment technologies. To improve the corrosion and tribological properties of stainless steel, Al_2_O_3_ [21,22,23], TiO₂ [24,25], or a combination of both oxides in the form of nanolaminates [26,27,28,29] are commonly used. Coatings consisting of nitrides [13] or oxides of other metals [30] are also used, but in this case, deterioration of the discussed properties is often observed. Therefore, the authors of this paper, considering the very good anti-corrosion properties of the TiO_2_ phase, decided to investigate the so-called bimodal TiO₂/nanoTiO₂ coating. The presented work aims to investigate the influence of the TiO_2_/nanoTiO_2_ coating process conditions obtained by a hybrid process combining magnetron sputtering (MS-PVD) and atomic layer deposition (ALD) methods on the structure and selected properties of these coatings.

## 2. Materials and Working Concept

**Figure 1:**
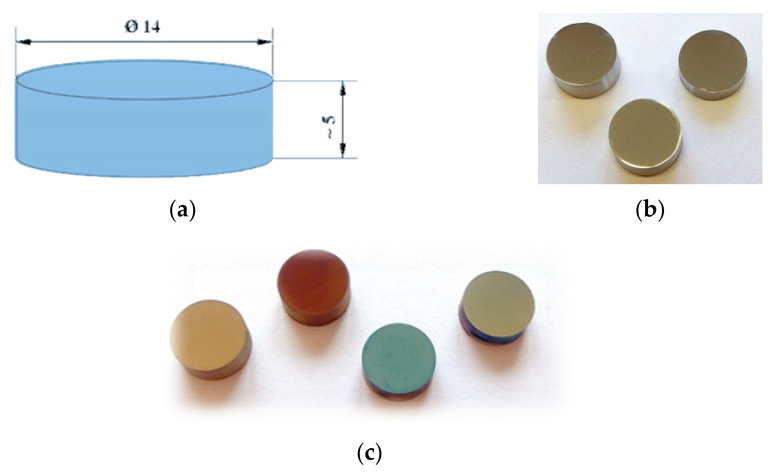
Test material: (**a**) sample dimensions, (**b**) uncoated substrates, and (**c**) samples with tested ALD coatings.

Tests were conducted on uncoated and coated Cr-Ni-Mo austenitic steel specimens. The samples were ground and polished and cleaned in an ultrasonic cleaner in acetone before coating. The dimensions of the specimens are shown in Figure 1, and their chemical compositions are shown in Table 1.

**Figure 2:**
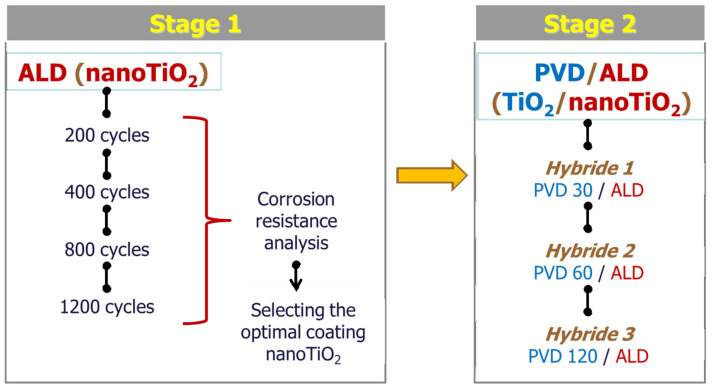
The concept of work.

The conceptual approach and optimization of the deposition conditions of the individual PVD and ALD layers was a two-step process. The first stage was concerned with optimizing the conditions for ALD deposition of titanium oxide. The highest corrosion resistance was the criterion for optimizing the ALD deposition process. Then, bimodal coatings were fabricated by PVD + ALD methods, in which the PVD layer deposition conditions were changed, while the ALD layer was deposited with previously optimized parameters (Figure 2).

### 2.1. Coatings Made by ALD Process

Coatings were deposited using the Thermal ALD method in an ALD PICOSUN R-200 flow reactor (Masala, Finland). The precursors were titanium tetrachloride and water. The variable parameters were the number of cycles. Four variants of coatings were performed. The coatings were applied at different numbers of ALD cycles (Table 2). The process temperature was fixed and was set at 200 °C. It was selected based on previous experience and research published in [31]. The deposition conditions are shown in Table 2. The titanium precursor was TiCl_4_ and the oxygen was H_2_O.

### 2.2. Hybrid Coatings

Hybrid coatings were deposited by depositing an optimized ALD layer on samples with PVD coatings applied (Kurt J Lesker PVD 75, Clairton, PA, USA). The hybrid coating combinations are shown in Table 3.

## 3. Methodology

The structure study was performed using a ZEISS Supra 35 microscope (Zeiss, Oberkochen, Germany). The imaging method used was secondary electron detection (SE) and an intra-lens detector. Analysis of the chemical composition in the microspheres was performed using the EDS method. The accelerating voltage was in the range of 5–20 kV.

A Park System XE-100 (Suwon, Korea) atomic force microscope was used to study the morphology of the studied samples. A non-contact mode was used with a probe elastic constant of 40 N/m and a resonance frequency of 300 kHz.

The electrochemical properties were determined by the following methods: potentiodynamic and electrochemical impedance spectroscopy. Tests were performed using Atlas Sollich 0531EU (Rębiechowo, Poland) potentiostat/galvanostat in 3.5% NaCl aqueous solution and Ringer’s solution at room temperature (23 °C) or temperature-simulating conditions in the human body (37 °C). The corrosion tests were performed in a three-electrode system, in which the reference electrode was an Ag/AgCl electrode with a potential of 207 mV, and the auxiliary electrode was made as a stainless steel wire. The corrosion resistance tests were performed in two steps:

- Determination of the open circuit potential (E_ocp_) by 1;
- Potentiodynamic method in the potential range E_start_ = E_ocp_ − 100 mV to E_finish_ = E_ocp_ + 100 mV, potential rise rate 1 mV/s

Characteristic electrical quantities describing corrosion resistance, i.e., current density (J_cor_) and corrosion potential (E_cor_) as well as polarization resistance (R_pol_) were determined using the Tafel method and AtlasLab software (Version 2.24, Atlas Sollich, Banino, Poland).

The electrical properties of the materials were also determined by a second impedance spectroscopy method, first by stabilizing the samples in the test environment for 15 min without current flow and then with forced AC flowing through the solidified system at an amplitude of 10 mV in the frequency range from 100 kHz to 10 mHz. The results are presented as Nyquist and Bode plots. To accurately represent the relationships appearing in the studied electrochemical process, an electrical equivalent circuit was assigned to them using AtlasLab and EC-Lab software (Version 11.20, BioLogic Science Instruments, Seyssinet-Pariset, France). The numerically generated curves were then matched to those recorded in the experiment and Constant Phase Element (CPE) nonlinear elements were used in addition to typical resistors.

Abrasion resistance tests were performed using the ball-on-disc method. The counterexample used was a WC-Co carbide ball with a diameter of 6 mm. The tests were performed at room temperature under the following test conditions: diameter 6 mm, linear velocity v = 0.5 cm/s, and normal force F_n_ = 0.5 N. The tests were performed with 500 cycles, where one cycle is one full rotation of the test specimen around its axis. The friction coefficient as a function of the number of cycles was the parameter recorded.

The structure of coatings on the cross-section was examined using a scanning-transmission electron microscope S/TEM Titan 80–300 made by FEI (Eindhoven, The Netherlands). Investigations of so-called lamellae were performed in both scanning and transmission modes at an applied accelerating voltage of 300 kV. The analysis of the chemical composition in the microspheres was performed using EDS and EELS spectroscopy. Phase analysis was performed by electron diffraction.

## 4. Results and Discussion

**Figure 3:**
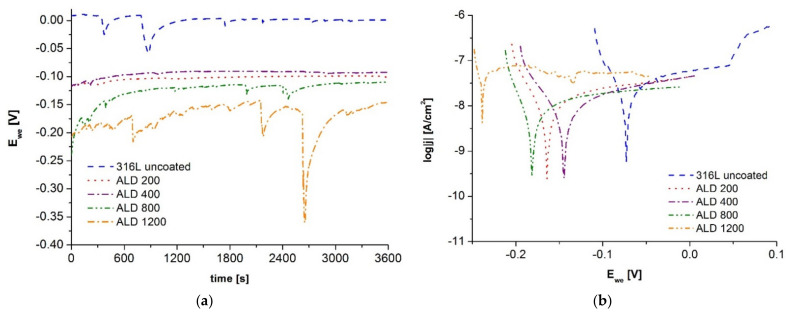
Electrochemical test curves of ALD coated materials: (**a**) open circuit potential, (**b**) anodic polarization curves.

The evaluation of the degree of corrosion resistance first consisted of corrosion tests of ALD-coated samples at varying numbers of process cycles. The tests were performed in Ringer’s solution at 37 °C. The open-circuit potential was determined under current-free conditions (Figure 3a), and in the next step, potentiodynamic polarization curves in the cathodic and anodic range were recorded for Tafel analysis (Figure 3b), the results of which are shown in Table 4.

All the samples with ALD coatings applied showed similar stationary potential values, albeit lower than the 316L steel substrate material sample, which may indicate the deteriorating stability in the studied environment for the coated samples.

Comparing the results obtained from the Tafel analysis, it can be concluded that the application of ALD coatings on the steel substrate resulted in an improvement in the resistance of the tested materials as indicated by an increase in polarization resistance from 2.6 to 3.5 times, the exception being the sample with the coating obtained after 1200 cycles, for which higher values of corrosion potential and current density were recorded compared to all other samples. The improvement in the resistance to the harmful effects of the tested environment of Ringer’s solution for the samples coated with ALD is also confirmed by the results of the value of the corrosion current density, which decreased to about 22 ÷ 38% of the value for the sample made of 316L steel.

The TiO_2_ coating obtained by PVD at 120 min was examined for comparison. Table 5 summarizes the results of the Tafel analysis performed on the basis of the potentiometric test in Ringer’s solution. From the study, the PVD coating shows a slight improvement in corrosion resistance, but it should be noted that its effect on corrosion resistance is small.

**Figure 4:**
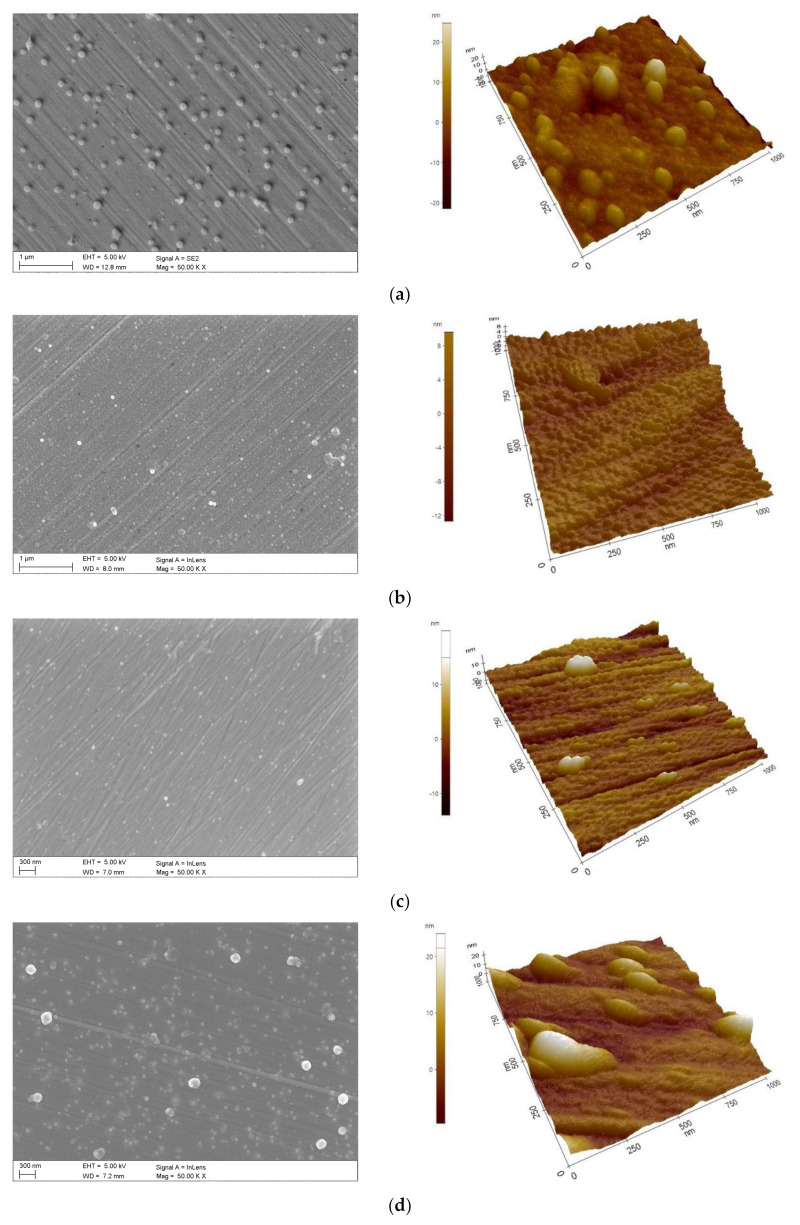
Morphology of ALD coatings (SEM, AFM): (**a**) ALD 200, (**b**) ALD 400, (**c**) ALD 800, (**d**) ALD 1200.

Observation of the surfaces of the investigated coatings by scanning electron microscopy revealed that the obtained layers are homogeneous, without pores and discontinuities. The morphology of the coatings reflects the morphology of the substrate, in particular, the scratches that remain after polishing the substrate before coating (Figure 4).

The atomic force microscope examination of the coating surfaces revealed that the ALD 400 and ALD 800 coatings exhibit the highest homogeneity. The layers obtained at the lowest and highest number of ALD 200 and ALD 1200 cycles, respectively, exhibit some inhomogeneity reflected by the AFM images and higher surface roughness than ALD 400 and 800 (Figure 4, Table 6).

**Figure 5:**
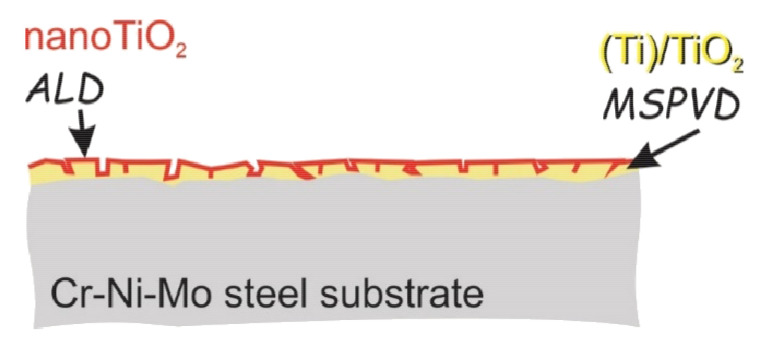
The idea of TiO_2_/nanoTiO_2_ bimodal coatings obtained by PVD/ALD hybrid process.

The general idea of bimodal coatings is shown in Figure 5. The first layer obtained by the PVD method provides high adhesion of the coating to the substrate, and in addition, the synergy obtained from the combination of these two technologies will allow obtaining a coating with physicochemical properties better than those of the layers obtained by each technology separately).

The table shows the conditions for obtaining the hybrid coatings. In the PVD process, the parameter was the deposition time. Three variants of the coating were made during deposition lasting half an hour, one hour, and two hours. Another titanium oxide layer was deposited on so-prepared titanium oxide layers by the ALD method in ALD 400 process optimized earlier. The coatings were designated successively as Hybrid 1, Hybrid 2, and Hybrid 3 (Table 3).

**Figure 6:**
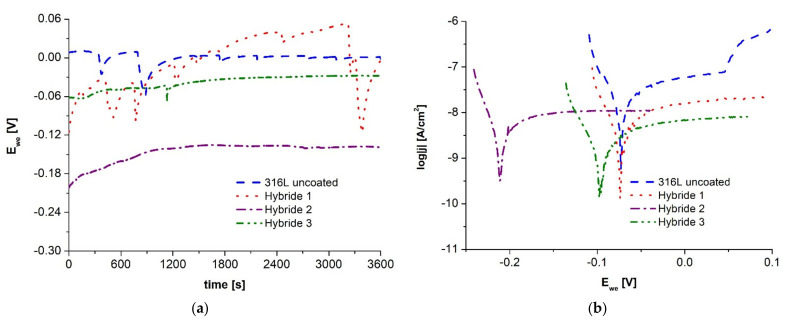
Electrochemical testing results of PVD + ALD hybrid coated materials: (**a**) open circuit potential, (**b**) anodic polarization curves in the range for Tafel analysis.

Corrosion resistance tests for bimodal coated materials were performed using identical conditions and sequential steps as for ALD coated samples. First, the three-electrode system was set up, and the steady-state potential was recorded for one hour (Figure 6a). Then, the anodic polarization curves were read from E_ocp_ − 100 mV to E_ocp_ + 100 mV for Tafel analysis (Figure 6b).

Based on the curves of the open-circuit potential, it can be observed that although its highest value was obtained for the sample with the coating applied for 30 min, clear jumps in the value of the potential may indicate its instability in the studied environment, which was not recorded for the samples after 60 and 90 min of the coating application process. The value of the free potential of the tested materials varied within a small range from −150 to −50 mV, and the highest value combined with a stable reading was recorded for the Hybrid 3 sample.

Analyzing the characteristic values determined by Tafel’s method, it can be stated that the materials coated with hybrid coatings were characterized by better corrosion resistance in comparison with the base material, which is evidenced by a higher value of polarization resistance and lower value of corrosion current density. The best corrosion resistance results in the studied environment were observed for the Hybrid 3 sample after 120 min PVD layer application process.

Additionally, the beneficial effect of the applied bimodal PVD + ALD 400 coatings was observed by comparing the electrochemical test results with samples coated with ALD coating only (Figure 6, Table 7). The hybrid sample maintained for the shortest coating time exhibited better anti-corrosion properties than the best of the samples after coating with ALD coating only, as evidenced by both lower current density values of the hybrid samples and higher polarization resistance values.

**Figure 7:**
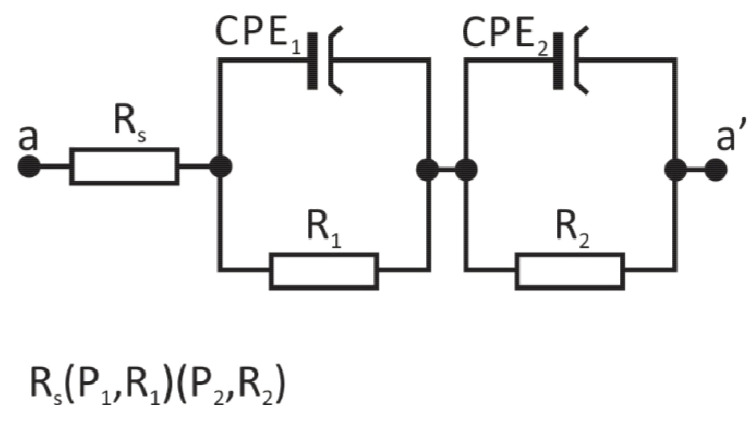
Electrical equivalent circuit for the investigated materials in Ringer’s solution.

To better characterize the electrochemical properties of the produced PVD + ALD coatings, impedance spectroscopy studies were performed for them. The use of the impedance method, which involves polarizing samples with an alternating voltage, allows very rapid results of the electrode response of the material under test. It is performed according to generally accepted procedures [32,33,34,35] at low voltage amplitude, in a wide range of frequencies and usually at equilibrium potential. The first step was to determine the equilibrium potential for each of the samples and then to record the impedance spectra in the frequency range of 100 kHz to 10 mHz, which were presented on the Nyquist and Bode diagrams. Based on the curves recorded during the test for the tested samples, an equivalent electrical circuit best describing the corrosion system was fitted, which consists of two CPE constant-phase elements (Figure 7), and its resultant impedance can be written with the following relation:

(1) $$Z=R_{s}+\frac{1}{\frac{1}{R_{1}}+Y_{1}{\left(j\omega \right)}^{\;n_{1}}}+\frac{1}{\frac{1}{R_{2}}+Y_{2}{\left(j\omega \right)}^{\;n_{2}}}$$

In the proposed electrical circuit, R_s_ is related to the resistance of the electrolyte or Ringer’s solution. R_1_ is the charge transfer resistance to the electrolyte from the surface zone of the material, that is, the first coating at the electrolyte, in this case, the ALD 400 coating. The CPE_1_ element models the capacitance of this zone. R_2_ is the charge transfer resistance across the phase boundary. The CPE_2_ element, on the other hand, can be thought of as reflecting the electrical properties of the double layer at the phase boundary.

**Figure 8:**
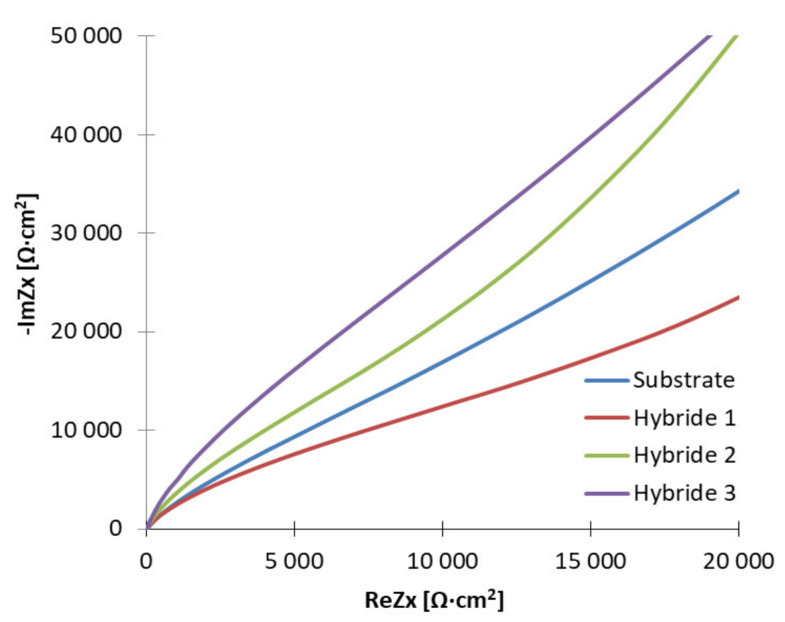
Impedance spectrum of the tested materials (for designated stationary potentials)—Nyquist representation.

Based on analyzing the shape of the curves from the Nyquist diagram (Figure 8), it can be concluded that in an attempt to simplify them into a linear function, the greater the value of the directional coefficient; in other words, the greater the value of the angle of inclination of such a curve, the greater the value of the resistance to electric current. Therefore, it is seen that increasing the time of application of PVD coatings to the substrate material favored the improvement of corrosion resistance of the produced material. The highest slope angle is for the sample with the hybrid coating after 120 min and the lowest for the coating applied for 30 min; i.e., the Hybrid 3 sample had the highest corrosion resistance in the tested environment, while the Hybrid 1 sample had the lowest (Table 8).

**Figure 9:**
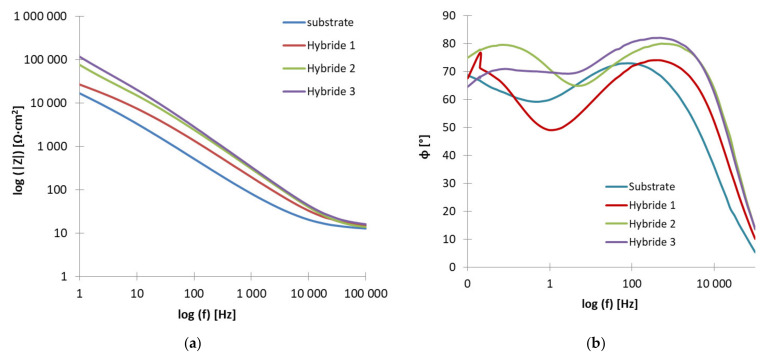
Impedance spectrum of the tested materials (for designated stationary potentials)—Bode representation: (**a**) impedance as a function of frequency, (**b**) phase angle as a function of frequency.

Presented in the form of a Bode plot, the impedance changes allow tracking the behavior of the corrosion system over a wide frequency range. The recorded curves show a characteristic course and decreasing impedance value with increasing frequency of the voltage signal (Figure 9a). The lowest impedance value in the whole range of the tested frequencies was found for the sample of the substrate material, while the highest impedance value was registered for the Hybrid 3 sample. In addition, the Bode plot shows a positive effect of prolonging the PVD coating process on the corrosion resistance of the material. The longer the period, the higher the impedance values obtained in the results.

The second type of Bode diagram (Figure 9b), showing the dependence of the phase shift angle on the impedance modulus, enables the evaluation of the quality or the tightness of the obtained coatings. In this case, the higher the value of the angle, the better the quality of the obtained sample, so the highest value can be evaluated for the Hybrid 3 coating material, the highest angle value of 82° can be read for it, and the lowest value was shown for the substrate material sample, about 71°. Similarly to the analysis of the previous results, it can be concluded that the prolongation of the PVD coating application process resulted in better coating quality, as evidenced by the increasing value of the phase shift angle.

It should be noted that the presented research is rather basic research, and possible applications are hypothetical at this stage of research. Thus, in addition to applications in biomedical engineering, such coatings could be used to coat, for example, structural details (components). Therefore, corrosion resistance tests were performed in a 3.5% NaCl solution. The test environment and temperature differ from previous corrosion tests. In this case, the following operating conditions of the EU potentiometer with a three-electrode measuring system (ATLAS-SOLLICH ATLAS 0531 EU) were used:

- Electrolyte: 3.5% NaCl
- Temperature: room temp.
- E_ocp_ during 1 h
- Potential growth rate 1 mV/s
- Electrodes: RE: Ag/AgCl; CE: steel; WE: samples

**Figure 10:**
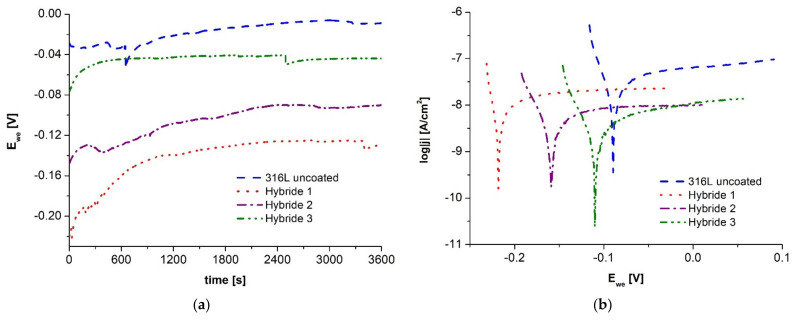
Electrochemical test curves of hybrid coated materials in 3.5% NaCl solution: (**a**) open circuit potential, (**b**) anodic polarization curves in the range for Tafel analysis.

The results of the recorded open circuit potential and potentiodynamic test curves in an aqueous solution of 3.5% NaCl are shown in Figure 10. For all three materials with hybrid coatings, the stability of the potential values in the tested environment was found, the value of which increased with increasing PVD coating deposition time from about −150 mV for the material with the coating obtained after 30 min to about −50 mV after 120 min of deposition.

The analysis of the results obtained from the Tafel calculations (Table 9) allows us to conclude that the application of hybrid coatings on the steel substrate caused a favorable change in the corrosion resistance of the tested materials, which is evidenced by an increase in the polarization resistance from 0.5 MΩ·cm^2^ for the substrate material to 1.2 MΩ·cm^2^ for Hybrid 1 sample, 3.9 MΩ·cm^2^ for Hybrid 2 sample and 10 times for Hybrid 3 sample. A similar relationship was noted by comparing the corrosion current density results, the lowest value of which was 1.2 nA/cm^2^ for the Hybrid 3 sample, decreased when shortening the PVD coating application time to 2.2 nA/cm^2^ for the Hybrid 1 sample but was still more than 4× higher than for the 316L steel substrate material.

**Figure 11:**
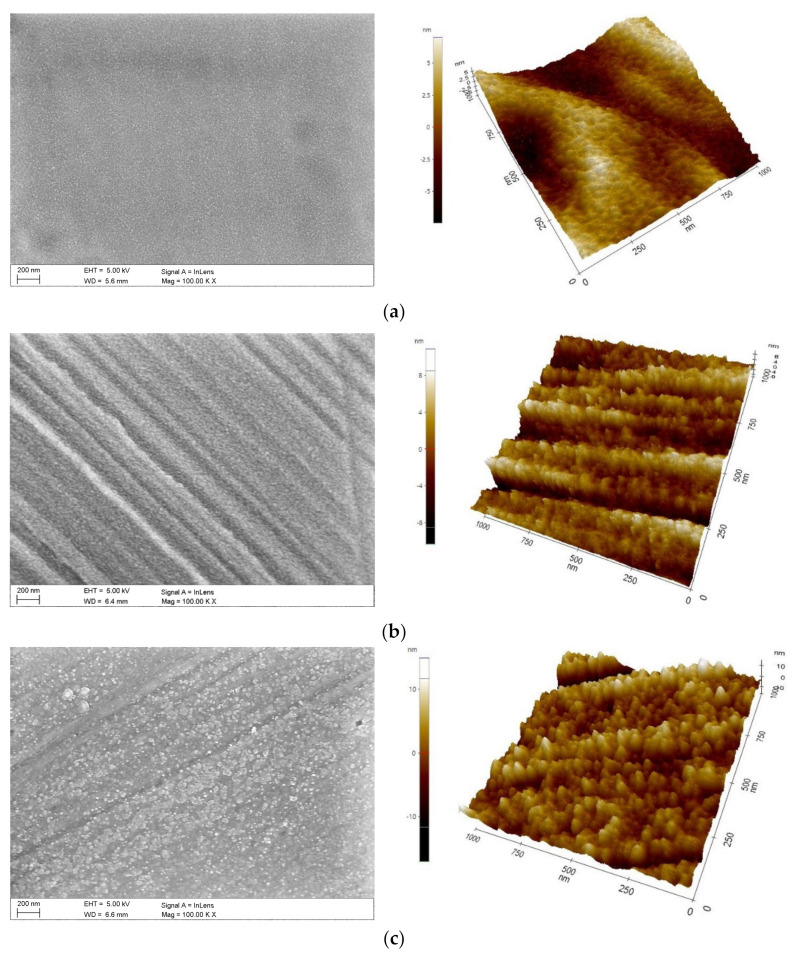
Morphology of PVD/ALD coatings (SEM, AFM): (**a**) Hybrid 1, (**b**), Hybrid 2, (**c**) Hybrid 3.

**Figure 12:**
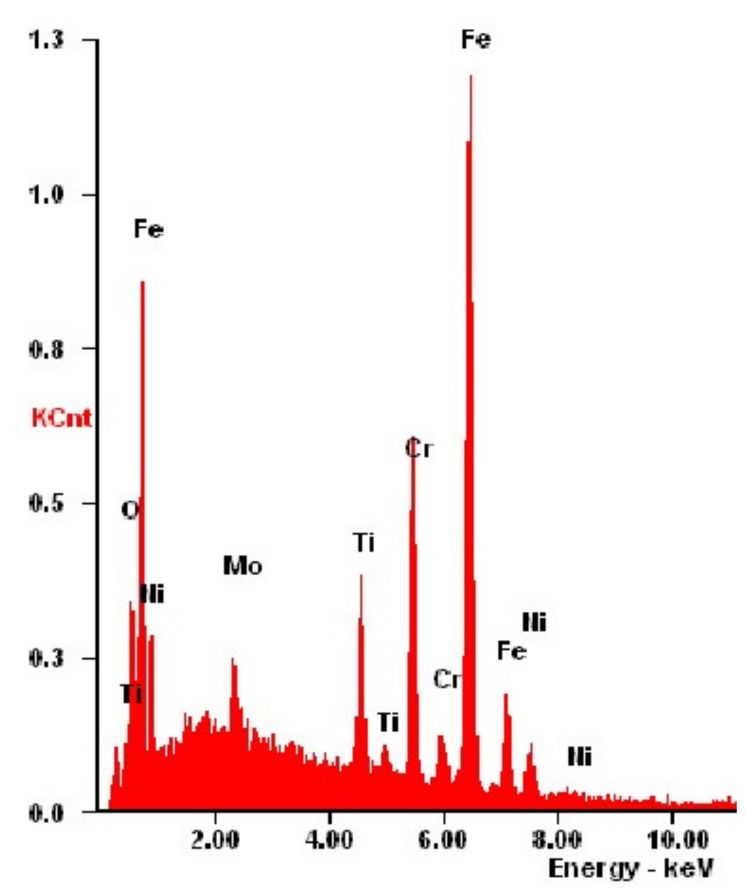
X-ray energy-dispersive plot of the area shown in Figure 11c.

Examination of the morphology (Figure 11, Table 10) of the bimodal coatings showed that the coatings have a compact structure without pores or discontinuities. Cracks are visible on the surface, which are residues from the substrate grinding process. The structure of the studied coatings in the morphological images consists of grains of different sizes. The smallest grains are shown by the TiO_2_/nanoTiO_2_ layer for which the PVD layer of titanium oxide was obtained in the shortest time of 30 min. (Hybrid 1). The largest grains are shown by the Hybrid 3 coating in which the PVD layer was deposited for 2 h. Furthermore, chemical composition analysis by EDS confirmed the presence of titanium and oxygen elements suitable for bimodal layers. Moreover, peaks from the steel substrate dominate the spectrograms (Figure 12).

**Figure 13:**
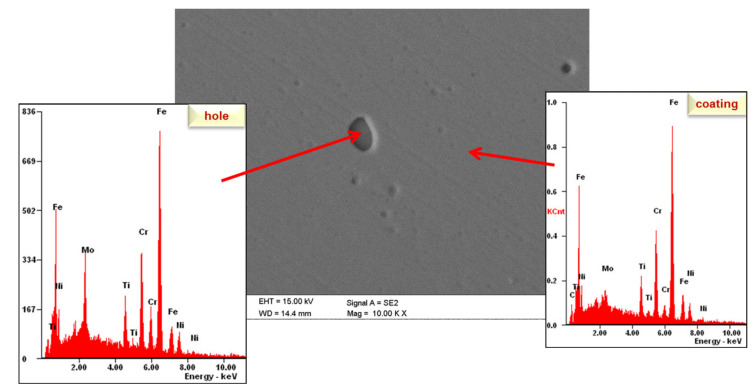
Corrosion mechanisms—SEM observations.

Observation of the coating surface after corrosion testing revealed small corrosion pits. EDS analysis confirmed the presence of a titanium element in the area outside the pitting, indicating the presence of a coating at this location. However, EDS analysis from the pitted areas also confirms the presence of a titanium peak, which is undoubtedly due to the excitation region also covering the coating area (Figure 13).

**Figure 14:**
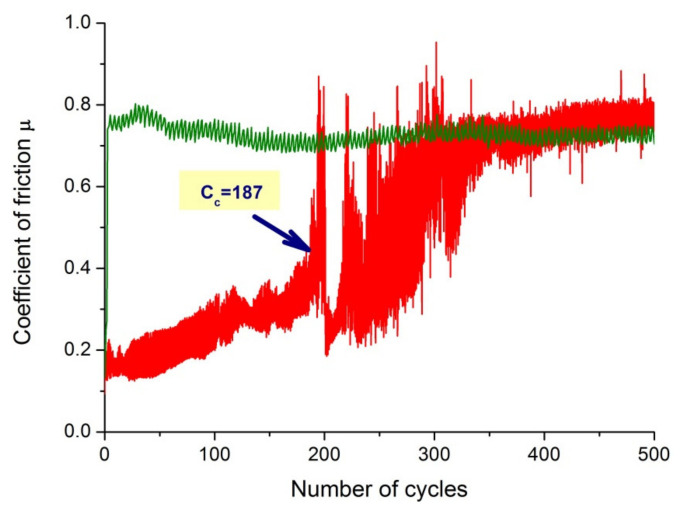
Friction coefficient as a function of the number of cycles for uncoated 316L steel substrate material and TiO_2_/nanoTiO_2_ (Hybrid 3) coating obtained by hybrid method.

**Figure 15:**
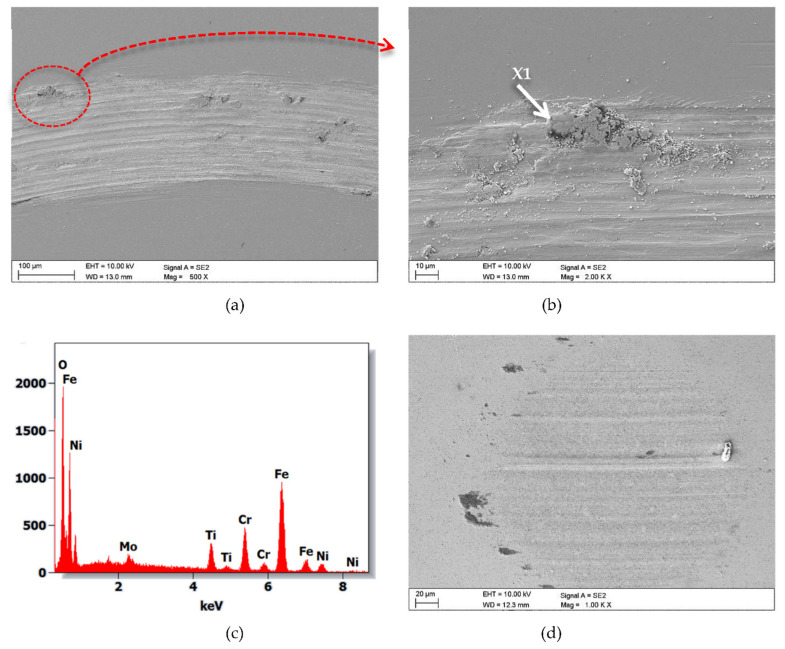
(**a**,**b**) Wear trace after the “ball-on-disc” wear test for TiO_2_/nanoTiO_2_ (Hybrid 3) bimodal coatings, (**c**) X-ray energy dispersive plot the area X1 as in Figure b, (**d**) wear place after the “ball-on-disc” wear test for cemented carbides ball as counter-sample.

Figure 14 shows plots of the friction coefficient µ as a function of the number of cycles for an uncoated steel specimen and one coated with a hybrid TiO_2_/nanoTiO_2_ bimodal coating. The friction coefficient plot for the uncoated sample is approximately µ = 0.75 and is close to the theoretical value for the steel-sintered carbide friction pair. In the case of a sample coated with a bimodal coating, the coefficient of friction initially did not exceed a mean value of 0.2 and increased to approximately µ = 0.4 during the test. As a result of scanning electron microscope observations, abrasion was found to be the main wear mechanism of both the tested samples and carbide counterexamples. Furrows were also found to be present (Figure 15). Moreover, the adhesive build-up of abraded coating material was found in different places of the wear path, which is confirmed by the presence of titanium and oxygen reflections on the EDS spectra from this area (Figure 15b,c).

**Figure 16:**
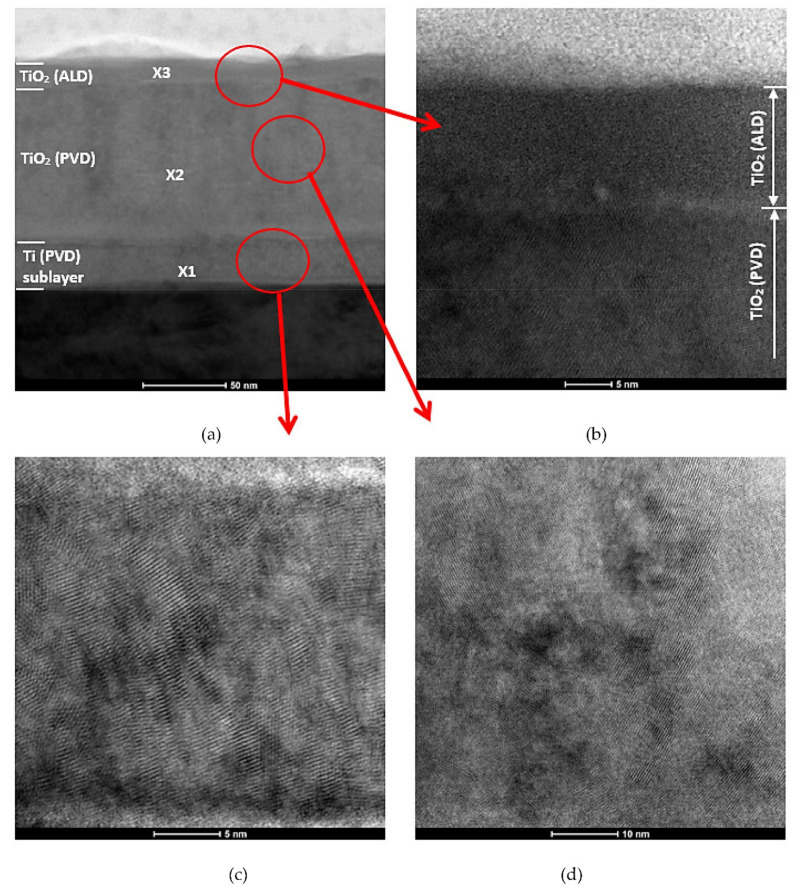
Structure (STEM) of TiO_2_/TiO_2_ bimodal coating: (**a**) structure of cross-section coating, (**b**) structure of TiO_2_ (ALD) layer and PVD / ALD transition zone, (**c**) structure of Ti sublayer (PVD), (**d**) structure of TiO_2_ (PVD) layer.

**Figure 17:**
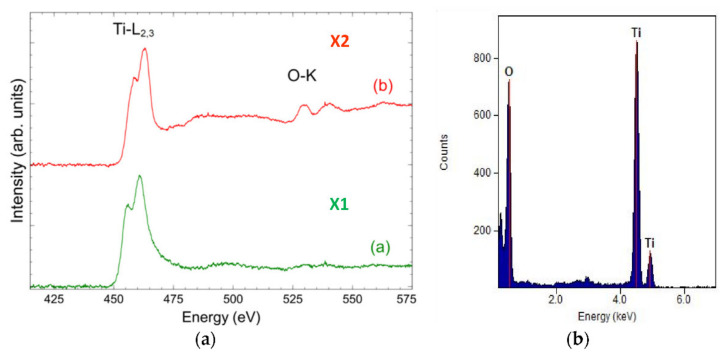
(**a**) EELS energy loss spectrum from areas X1, X2, and X3 according to Figure 16, (**b**) X-ray energy-dispersive plot of the area X3 shown in Figure 16.

**Figure 18:**
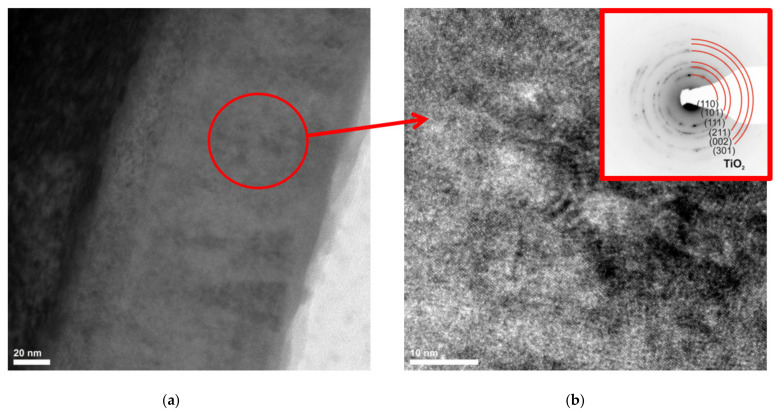
Structure (TEM) of TiO_2_/TiO_2_ bimodal coating obtained: (**a**,**b**) bright field (b with diffraction pattern).

Based on the examination of a thin film from a cross-section of a bimodal TiO_2_/TiO_2_ coating by transmission electron microscopy, it was confirmed that the coating exhibits a layered structure (Figure 16). The layers are discussed sequentially starting from the substrate. The thicknesses of each layer are 28 nm, 93 nm, and 15 nm. EELS and EDS investigations of the chemical composition of the micro-areas confirm the presence of elements appropriate for individual layers, i.e., titanium for the first layer and titanium and oxygen for the second and third layers (Figure 17). The first two layers obtained by MSPVD show features of crystalline structure. Moreover, diffraction analysis confirmed the presence of the TiO_2_ phase with tetragonal structure P42/mnm in the second layer (Figure 18). The third layer is also titanium oxide, as confirmed by chemical composition analysis. Despite this, however, it shows clear characteristics of an amorphous phase.

## 5. Summary

The importance of coating technology as a technology for shaping the structure and surface properties of engineering materials, including PVD and ALD methods, is increasing in modern industry due to the numerous possibilities of application of these technologies to different groups of engineering materials and their high efficiency, ecological advantages, and economic justification.

**Figure 19:**
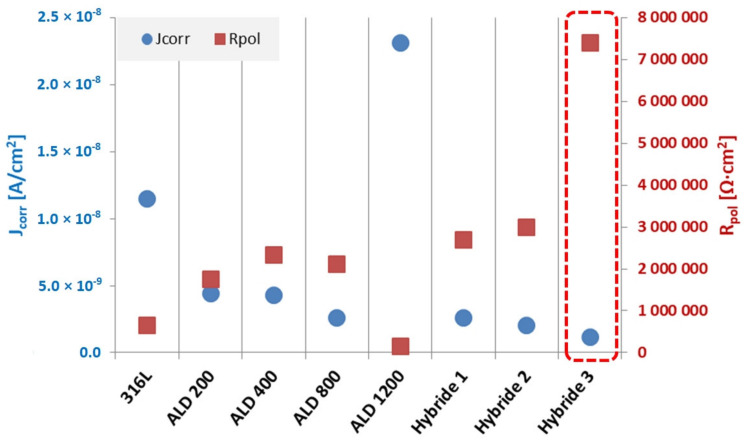
Summary of corrosion parameters values from Tafel analysis of samples with ALD and PVD+ALD coatings in Ringer’s solution.

The best anti-corrosion properties are provided by bimodal TiO_2_/nanoTiO_2_ coating obtained by hybrid PVD + ALD process designated as Hybrid 3. The applied coatings, both by the ALD process and the combination of PVD and ALD methods, improved the corrosion resistance of the tested 316L steel in electrochemical tests. For the best of the samples after the ALD process, the corrosion current decreased by 77% compared to the substrate material, and for the samples with the hybrid coating, there was an even greater improvement as the corrosion current density decreased by 85% (Figure 19).

This is because the nanoTiO_2_ layer with amorphous structure characteristics obtained by the ALD technique tightly covers all imperfections (nano cracks, chipping) of the TiO_2_ coating, which is a barrier to the development of corrosion. The very good corrosion resistance of TiO_2_ layers deposited by ALD technology was also confirmed by the authors in works [24,25,36]. The morphology of the studied coatings shows a granular structure with variable grain size. The PVD layer is responsible for the granularity of these coatings because, as shown by TEM studies of thin films on cross-section, this layer exhibits a crystalline structure. The ALD layer, on the other hand, exhibits an amorphous structural structure with a small thickness. It also accurately reproduces the morphology of the preceding PVD layer.

**Figure 20:**
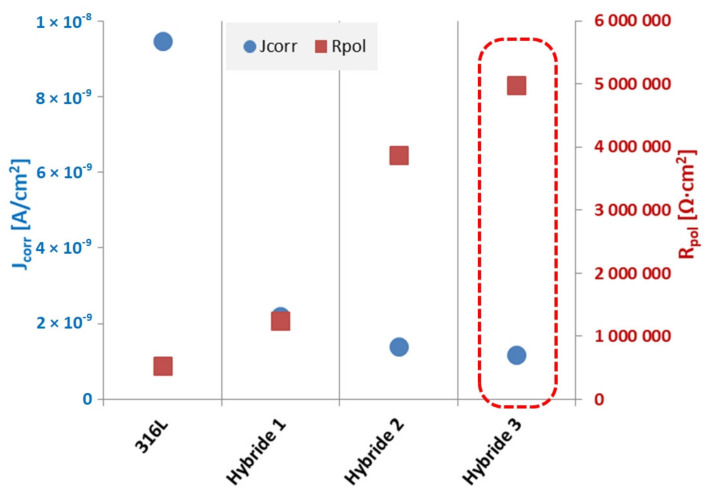
Summary of corrosion parameters values from Tafel analysis of samples with hybrid coating in 3.5% NaCl solution.

Similarly, as in the case of corrosion resistance test in Ringer’s solution, the applied hybrid coatings in NaCl word solution significantly improved the properties of the substrate material, for both characteristic electrochemical parameters an improvement was found: the corrosion current density decreased by almost 8 times and polarization resistance by 10 times in comparison with the substrate material (Figure 20).

Sealing the PVD coating with an ALD coating designated as Hybrid 3 also resulted in improved tribological properties. As a result of the application of the bimodal TiO_2_/TiO_2_ coating on the substrate made of austenitic steel, a very low coefficient of friction ranging from 0.2 to 0.4 was observed in the first phase of the test until the critical number of cycles was reached. The lower coefficient of friction and thus low wear of the hybrid bimodal TiO_2_/TiO_2_ coating should be attributed to compensating any deficiencies of the PVD coating through the strengthening effect of the ALD layer sealing it. This layer largely reduced areas that could have been the origin of crack initiation and chipping.

The excellent corrosion resistance and improved tribological properties obtained by the TiO_2_/TiO_2_ bimodal coating are closely related to the obtained hybrid structure, which, as shown by cross-sectional studies using S-TEM, consists of two layers with crystalline features and a tetragonal lattice, which are sealed by an ALD layer with the features of an amorphous phase. The formation of the amorphous structure is related to the number of cycles of the ALD process. A lower number of cycles favors the formation of the amorphous structure of the TiO_2_ phase. This was also confirmed in the research work [32].

## 6. Conclusions

Based on the performed studies, it can be concluded that:

- The use of a hybrid process combining PVD and ALD methods for the deposition of bimodal TiO_2_/nanoTiO_2_ coatings makes it possible to obtain, by synergy, high corrosion resistance of the coated 316L steel impossible to obtain using each coating technique separately.
- An improvement in the corrosion resistance of the tested materials was found in both Ringer’s solution and an aqueous solution of 3.5% NaCl. The greatest improvement was observed for Hybrid 3. In this case, the corrosion current density decreased by 85%. Using sodium chloride solution, the corrosion current density decreased eightfold. On the other hand, the polarization resistance value increased 10-fold compared to the substrate material.
- The tested coatings show an effect on reducing the friction coefficient in the first phase of the test to reach a critical number of cycles and thus improving the tribological contact in the case of Hybrid 3.
- TiO_2_ layer obtained by the PVD technique shows crystalline structure while the titanium oxide layer deposited by the ALD process is amorphous.

In conclusion, as a result of the performed research, we recommend Hybrid 3 coating. It provides improved tribological properties and very high corrosion resistance.

## Figures and Tables

**Table 1:** Chemical composition of X2CrNiMo17–12–2/1.4404 steel.

Elemental Concentration [%]							
C	Cr	Ni	Mn	Mo	N	P	S
0.006	18.3	10.7	1.67	2.11	0.07	0.04	0.02

**Table 2:** Deposition conditions of ALD coatings.

Temperature (°C)	Reagent Feed Time (s)		Cleaning Time (s)		Number of Cycles
	TiCl_4_	H_2_O	After TiCl_4_	After H_2_O	
200	0.1	0.1	4	5	200
					400
					800
					1200

**Table 3:** Conditions of deposition of hybrid coatings.

		Hybrid 1	Hybrid 2	Hybrid 3
PVD (TiO_2_)	Metal vapour source (target)	Ti		
	Gases	N_2_, O_2_		
	Temperature proc. (°C)	100		
	Ti layer deposition time (min.)	2		
	Deposition time of TiO_2_ layer (min.)	30	60	120
	Power on magnetron source (W)	200		
	BIAS (V)	140		
ALD (TiO_2_)	Titanium source	TiCl_4_		
	Oxygen source	H_2_O		
	Temperature (°C).	200		
	Number of ALD cycles	400		

**Table 4:** Summary of corrosion parameter values from Tafel analysis of ALD coated samples.

	316L	ALD 200	ALD 400	ALD 800	ALD 1200
E_cor_ (V)	−0.073	−0.164	−0.144	−0.181	−0.239
J_cor_ (nA/cm^2^)	11.5	4.4	4.3	2.6	23.1
R_pol_ (MΩ∙cm^2^)	0.66	1.75	2.33	2.1	0.14

**Table 5:** Summary of corrosion parameter values from Tafel analysis of PVD coated samples.

	316L	TiO_2_ (PVD 120 min)
E_cor_ (V)	−0.073	−0.136
J_cor_ (nA/cm^2^)	11.5	5.7
R_pol_ (MΩ∙cm^2^)	0.66	0.69

**Table 6:** Summary of coating roughness obtained by the ALD process.

Roughness R_a_ (nm)			
ALD 200	ALD 400	ALD 800	ALD 1200
6.03	2.76	2.46	8.53

**Table 7:** Summary of corrosion parameter values from Tafel analysis of hybrid coated samples in Ringer solution.

	316L	Hybrid 1	Hybrid 2	Hybrid 3
E_corr_ (V)	−0.073	−0.073	−0.218	−0.097
J_corr_ (nA/cm^2^)	11.5	2.6	2.0	1.1
R_pol_ (MΩ∙cm^2^)	0.7	2.7	2.9	7.4

**Table 8:** Electrical equivalent circuit values of the tested hybrid samples.

Material	R_s_ (Ω·cm^2^)	Y_1_ (µS·cm^2^)	N_1_	R_1_ (kΩ·cm^2^)	Y_2_ (µS·cm^2^)	N_2_	R_2_ (MΩ·cm^2^)
361L	6.2	22.7	0.775	6.2	19.4	0.859	10.8
Hybrid 1	6.6	14.9 × 10^−6^	0.805	6.8	4.7 × 10^−6^	0.847	14.9
Hybrid 2	6.2	2.7 × 10^−6^	0.889	6.3	2.2 × 10^−6^	0.926	10.4
Hybrid 3	7.0	2.2 × 10^−6^	0.865	7.2	1.9 × 10^−6^	0.997	23.8

**Table 9:** Summary of corrosion parameter values from Tafel analysis of hybrid coated samples in 3.5% NaCl solution.

	316L	Hybrid 1	Hybrid 2	Hybrid 3
E_corr_ (V)	−0.089	−0.219	−0.159	−0.108
J_corr_ (nA/cm^2^)	9.5	2.2	1.4	1.2
R_pol_ (MΩ·cm^2^)	0.5	1.2	3.9	5.0

**Table 10:** PVD + ALD bimodal coatings roughness summary.

Roughness R_a_ (nm)		
Hybrid 1	Hybrid 2	Hybrid 3
2.3	2.2	2.8

## Data Availability

The data presented in this study are available on request from the corresponding author.
